# Role of phlebotomy in the treatment of liver damage related to erythropoietic porphyria

**DOI:** 10.1038/s41598-022-10089-z

**Published:** 2022-04-12

**Authors:** Satoru Hagiwara, Naoshi Nishida, Hiroshi Ida, Kazuomi Ueshima, Yasunori Minami, Masahiro Takita, Tomoko Aoki, Masahiro Morita, Hirokazu Chishina, Yoriaki Komeda, Akihiro Yoshida, Ah-Mee Park, Masako Sato, Akira Kawada, Hajime Nakano, Hiroshi Nakagawa, Masatoshi Kudo

**Affiliations:** 1 Department of Gastroenterology and Hepatology, Kindai University Faculty of Medicine, 377-2 Ohno-Higashi, Osaka-Sayama, Osaka, 589-8511 Japan; 2 Department of Microbiology, Kindai University Faculty of Medicine, Osaka, Japan; 3 Department of Dermatology, Kindai University Faculty of Medicine, Osaka, Japan; 4 Department of Dermatology, Hirosaki University Hospital, Hirosaki, Japan; 5 Department of Biological Chemistry, College of Bioscience and Biotechnology, Chubu University, Kasugai, Japan

**Keywords:** Gastroenterology, Pathogenesis

## Abstract

Liver damage affects the prognosis of patients with erythropoietic protoporphyria (EPP). However, there is no radical cure for EPP patients with severe liver damage. This study aims to investigate the effectiveness of phlebotomy in patients with severe liver damage. We examined seven patients diagnosed with EPP and liver damage between 2010 and 2020. Of the 7 cases, phlebotomy was performed in 3 cases with severe hepatic disorder, and the improvement effect of hepatic disorder was observed in all cases. In addition, as an additional study, we also investigated the mechanism by which liver damage becomes more severe. Liver biopsy samples were stained with hematoxylin and eosin and immunohistochemistry was used to examine the expression of adenosine triphosphate-binding transporter G2 (ABCG2). Liver biopsies were performed in 3 of 7 patients with EPP. Of these three patients, ABCG2 expression was low in two patients, especially in the protoporphyrin (PP) deposition area. Two patients with reduced ABCG2 expression subsequently developed severe liver damage. However, the causal relationship between the decreased expression of ABCG2 and the exacerbation of liver damage has not been directly proved, and further investigation is required in the future. This study demonstrated the effectiveness of phlebotomy in EPP patients with severe liver damage.

## Introduction

Hereditary porphyria is a metabolic disorder that develops when any of the enzymes involved in the heme synthesis system is genetically impaired. This disease significantly impairs the patients’ quality of life; currently, there is no radical treatment for hereditary porphyria indicating that patients may develop serious sequelae, which may be fatal. Erythropoietic porphyria (EPP) is caused by mutations in the *FECH* gene^1^. In 10%–20% of patients with EPP, hepatic dysfunction is observed due to deposition of erythrocyte protoporphyrin and serum protoporphyrin in hepatocytes and bile canaliculi^2,3^. Approximately 2%–5% of patients with EPP die because of liver damage gradually progressing to cirrhosis or liver failure (chronic liver failure), which rapidly develops into irreversible cholestatic liver failure (acute liver insufficiency). Regarding the onset of liver dysfunction, in cases with mutations in the *FECH* gene that lead to loss of enzyme activity, the precursor protoporphyrin potentially accumulates, leading to an increased incidence of liver damage. In contrast, while it is reported that aggravation of liver damage correlates with protoporphyrin levels^4^, other study have reported cases without any correlation between liver damage and serum protoporphyrin levels^5^. Currently, our understanding about the association of liver damage with EPP is limited.

Ursodeoxycholic acid^6^, cimetidine^7–9^, cholestyramine^10^, and plasmapheresis^11^, among other compounds, have been reported to be effective for liver damage in EPP. Moreover, liver transplants have been reported to be beneficial; however, the long-term clinical course after treatment remains unclear^12^. Therefore, there is an urgent need to identify the most effective treatment for patients with severe liver damage.

In this study, we report the efficacy of phlebotomy in EPP patients with severe liver injury treated at our hospital.

## Results

### Patient background

The median age at referral was 31 years (26–64 years). Of the seven patients, six were male and one was female. Family history was observed in 6 patients and photosensitivity was observed in all 7 patients. The serum PP level at the time of referral was as high at 4059 (range 2973–14,518) µg/dL red blood cells (RBCs) (Table 1). EPP was confirmed in six patients by genetic testing (Table 2)^13^. All the EPP patients in which *FECH* mutations were identified are predicted to be caused by the pathogenic *FECH* mutation in combination with the low expression allele c.315-48C *in trans*^13^.

**Table 1:** Patient background.

No	Age at the first visit (years)	Sex	PP (μg/dL/RBC)	Family history	Photo-sensitivity	Genetic proof	Liver fibrosis by percutaneous liver biopsy (HAI scoring)	Treatment	Liver carcinogenesis	Prognosis
1	61	Female	14,518	Unknown	+	-	N.E	UDCA: 600 mg; cimetidine: 800 mg; phlebotomy; shading	-	Alive
2	26	Male	4020	+	+	+	Stage 1	Shading	-	Alive
3	27	Male	3952	+	+	+	N.E	UDCA: 600 mg; cimetidine: 800 mg; cholestyramine: 27 g; shading	-	Alive
4	64	Male	4059	+	+	+	N.E	Cimetidine: 800 mg; shading	-	Alive
5	28	Male	4883	+	+	+	Stage 4	UDCA: 600 mg; cimetidine: 800 mg; cholestyramine: 27 g; phlebotomy; shading	-	Alive
6	31	Male	10,175	+	+	+	Stage 1	UDCA: 600 mg; cimetidine: 800 mg; phlebotomy; shading	-	Alive
7	33	Male	2973	+	+	+	N.E	Shading	-	Alive

*PP* protoporphyrin, *UDCA* ursodeoxycholic acid, *HAI* histological activity index.

**Table 2:** Genetic testing for EPP diagnosis.

No	Mutations in FECH	c.315–48
1	N.E	N.E
2	c.1077+1G>A	c.315-48C/C
3	c.804+1886_c.1078-653delins54	c.315-48T/C
4	c.804+1886_c.1078-653delins54	c.315-48T/C
5	c.1077+1G>A	c.315-48C/C
6	c.67+2935_c.464-786del13103	c.315-48C/-
7	c.67+2935_c.464-786del13013	c.315-48C/-

*N.E.* not evaluated.

### Liver function and iron metabolism test at the time of referral

Table 3 shows the results of each liver function test at the time of referral. Hepatocellular injury type was noted in one patient (No. 6), cholestatic type in two (No. 1, 4), mixed type in one patient (No. 5), and an unclassifiable type in three patients (No. 2, 3, and 7)^14^. In addition, two patients showed jaundice at the time of referral (No. 1, 3). No obvious anemia was observed in 7 patients at the time of referral.

**Table 3:** Liver function and iron metabolism at the first visit.

No	PP (μg/dL/RBC)	AST (U/L)	ALT (U/L)	ALP (U/L)	γGTP (U/L)	T-bil (mg/dL)	Pattern of liver injury	Hb (g/dL)	Fe (μg/dL)	Ferritin (ng/mL)	Erythroblast
Normal range M (male) F (Female)	30–86	13–30	M 10–42 F 7–23	106–322	13–64	0.4–1.5		M 13.7–16.8 F 11.6–14.8	40–188	M 39.9–465 F 6.2–138	Appearance
1	14,518	108	106	774	132	11.3	Cholestatic	11.4	32	48	No appearance
2	4020	31	51	222	18	0.9	Unclassified	14.6	123	11	No appearance
3	3952	40	75	205	38	1.6	Unclassified	13	77	28	No appearance
4	4059	89	121	608	1325	0.8	Cholestatic	13.4	85	30	No appearance
5	4883	193	312	690	416	7	Mixed	12.6	64	73	No appearance
6	10,175	75	138	315	243	1.4	Hepatocellular	13.8	17	25	No appearance
7	2973	31	40	306	39	0.9	Unclassified	14.5	192	68	No appearance

*PP* protoporphyrin, *AST* aspartate aminotransferase, *ALT* alanine aminotransferase, *ALP* alkaline phosphatase, *γGTP* γ-glutamyl transpeptidase, *T-bil* total bilirubin, *Hb* hemoglobin.

### Treatment and prognosis

Thorough shading was performed in all the nine patients. Ursodeoxycholic acid was used in four patients, cimetidine in five patients, and colestyramine in two patients (Table 1). Since these existing treatments were ineffective, 3 patients (No1, No5, No6) subsequently added phlebotomy. No cases of liver cancer were observed during the course of the study until March 2021.

### Laboratory data immediately before phlebotomy and efficacy of phlebotomy

Blood chemistry data (collected immediately before phlebotomy) are summarized for the three patients (No. 1, 5, and 6) who underwent phlebotomy. All the three patients had overt jaundice and significantly elevated serum PP levels (Table 4).

**Table 4:** Laboratory data immediately before and after phlebotomy.

	Patient No.1 Before	Patient No.1 After	Patient No.5 Before	Patient No.5 After	Patient No.6 Before	Patient No.6 After
**Blood chemistry**						
TP	5.6 g/dL	6.8 g/dL	6.4 g/dL	7.1 g/dL	6.4 g/dL	7.2 g/dL
Alb	2.3 g/dL	3.5 g/dL	4.0 g/dL	4.9 g/dL	4.0 g/dL	4.3 g/dL
BUN	7 mg/dL	9 mg/dL	9 mg/dL	7 mg/dL	10 mg/dL	9 mg/dL
Cr	0.34 mg/dL	0.54 mg/dL	0.57 mg/dL	0.62 mg/dL	0.46 mg/dL	0.59 mg/dL
T-Bil	11.3 mg/dL	1.1 mg/dL	9.6 mg/dL	1.0 mg/dL	3.3 mg/dL	0.6 mg/dL
D-Bil	9.2 mg/dL	0.7 mg/dL	7.5 mg/dL	0.4 mg/dL	2.5 mg/dL	0.3 mg/dL
ALP	774 U/L	684 U/L	558 U/L	303 U/L	287 U/L	286 U/L
AMY	35 U/L	117 U/L	42 U/L	54 U/L	88 U/L	114 U/L
LDH	142 U/L	143 U/L	162 U/L	118 U/L	190 U/L	114 U/L
AST	108 U/L	25 U/L	122 U/L	23 U/L	245 U/L	21 U/L
ALT	106 U/L	19 U/L	260 U/L	26 U/L	175 U/L	16 U/L
γGTP	132 U/L	15 U/L	322 U/L	34 U/L	442 U/L	200 U/L
Ferrtin	32 ng/mL	11 ng/mL	114 ng/mL	6 ng/mL	60 ng/mL	3 ng/mL
CRP	1.806 mg/dL	0.023 mg/dL	0.059 mg/dL	0.018 mg/dL	0.149 mg/dL	0.054 mg/dL
PP	14,518 µg/dL	2162 µg/dL	5093 µg/dL	1354 µg/dL	10,198 µg/dL	2710 µg/dL
RBC-PP	6451 µg/dL	1549 µg/dL	3474 µg/dL	1027 µg/dL	6237 µg/dL	1919 µg/dL
**Coagulation**						
PT	67.1%	68.9%	94.0%	93.7%	114.7%	87.3%
INR	1.19	1.14	1.02	1.02	0.95	1.06

*TP* total protein, *Alb* albumin, *BUN* blood urea nitrogen, *Cr* creatinine, *T-Bil* total bilirubin, *D-Bil* direct bilirubin, *ALP* alkaline phosphatase, *AMY* amylase, *LDH* lactate dehydrogenase, *AST* aspartate aminotransferase, *ALT* alanine aminotransferase, *γGTP* γ-glutamyl transpeptidase, *CRP* C-reactive protein, *PP* protoporphyrin, *PT* prothrombin time, *INR* international normalized ratio.

**Figure 1:**
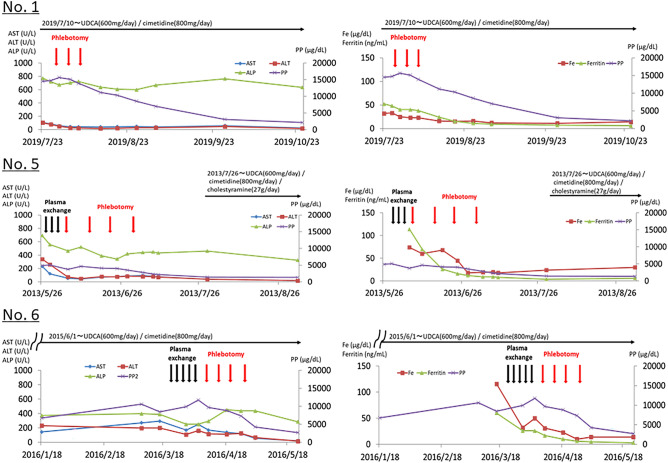
Clinical course after phlebotomy. In patient No. 1, plasma exchange was not performed. Phlebotomy therapy was performed three times (first time, 400 mL; following 2–3 times, 200 mL). In patient No. 5, a plasma exchange was performed five times; however, the serum PP level and liver damage did not improve; thus, the treatment was changed to phlebotomy. Phlebotomy was carefully performed four times in total (400 mL the first time; 200 mL thereafter). In patient No. 6, plasma exchange was performed four times; however, improvement in the serum PP level and hepatitis is not obtained. Thus, the treatment was changed to phlebotomy. Phlebotomy was performed four times in total (400 mL the first time; 200 mL thereafter).

In patient No. 1, plasma exchange was performed five times; although serum PP levels were decreased, hepatic injury did not improve. Phlebotomy was performed four times in total (400 mL was removed in the first time and 200 mL was removed thereafter). A decrease in the serum PP level and subsidence of liver injury was observed, along with a decrease in the Hb and serum ferritin levels (Fig. 1).

In patient No. 5, plasma exchange was performed five times and the serum PP levels were decreased; however, liver injury did not improve. Phlebotomy was conducted four times in total (400 mL was removed in the first time and 200 mL removed thereafter), and a decrease in the serum PP level was observed with improvement in liver injury (Fig. 1).

In patient No. 6, phlebotomy therapy was performed three times (400 mL was removed in the first time and 200 mL was removed thereafter) from the beginning; the serum PP level increased and liver injury improved along with a decrease in the Hb and serum ferritin levels (Fig. 1).

### Pathological findings

**Figure 2:**
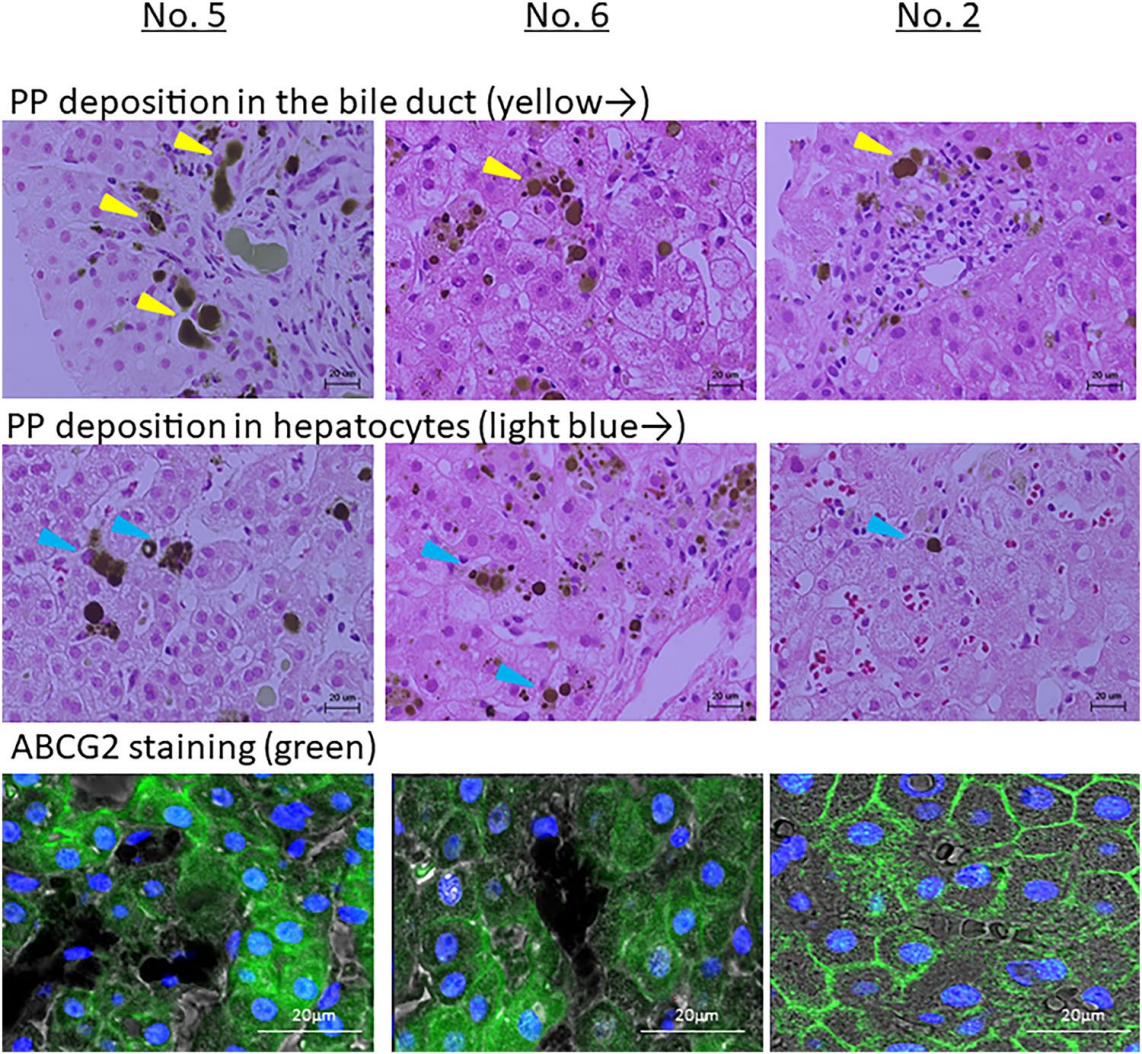
Hematoxylin and eosin (H&E) staining and ABCG2 staining pathology. Liver biopsy was performed in four patients, and the Maltese cross was confirmed in all patients. Biopsy samples were subjected to H&E and ABCG2 staining.

Liver biopsy was performed in 3 cases, and the Maltese cross was confirmed in all cases. In No. 5 and No. 6, PP deposits were distributed in the bile duct and hepatocytes. On the other hand, in No. 2, PP deposition was mainly in the bile duct and less was deposited in hepatocytes (Fig. 2). The stainability of ABCG2 was guaranteed in No. 2, On the other hand, it showed a marked decrease in No. 5 and No. 6 (Fig. 2).

**Figure 3:**
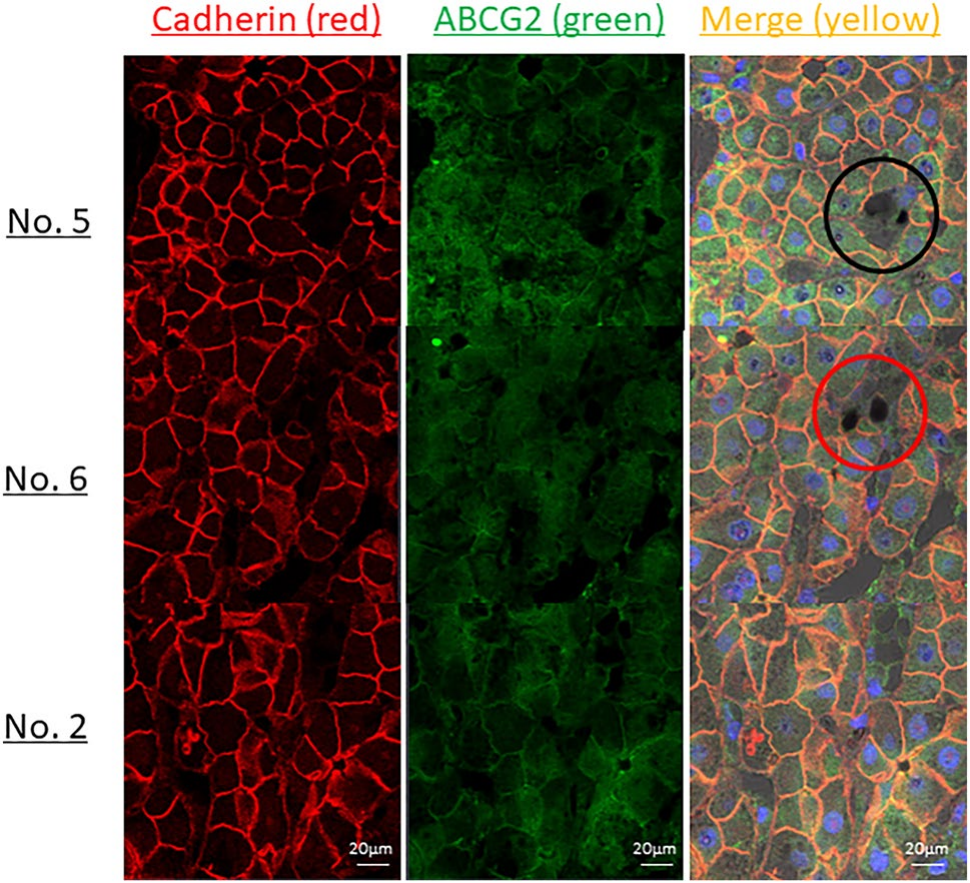
ABCG2 and cadherin double staining pathology. To evaluate the function of the hepatic cell membrane, cadherin staining was performed and double staining with ABCG2 was performed. In No. 5, cadherin (red) was strongly stained in PP-deposited hepatocytes, but ABCG2 (green) was weakly stained (black circles). In No. 6, both the staining properties of ABCG2 (green) and cadherin (red) were decreased in PP-deposited hepatocytes (the part indicated by the red circle). On the other hand, in No. 2, the stainability of ABCG2 and cadherin was guaranteed.

Moreover, to evaluate damage in the cell membrane of affected hepatocytes, double staining with ABCG2 and cadherin was performed (Fig. 3). In patient No. 5, cadherin was well expressed in PP-deposited hepatocytes, whereas ABCG2 was stained, suggesting the selective loss of ABCG2 activity on the cell membrane. Although the expression of cadherin in PP-deposited hepatocytes was heterogeneously detected in patient No. 6, ABCG2 staining was reduced in hepatocytes with PP deposition, indicating the loss of ABCG2 activity on the preserved cell membrane as well as damage to the cell membrane. Contrastingly, in patient No. 2, the expression of cadherin and ABCG2 was maintained.

## Discussion

In general, liver damage is a factor that mostly affects the prognosis of patients with EPP. Approximately 5–10% of all patients with EPP have liver damage, where 1% of these patients have fatal conditions^2,3^. Therefore, there is an urgent need to establish effective treatment for EPP patients with severe liver damage. EPP is caused by the accumulation of excess protoporphyrin in RBCs; hence, plasmapheresis may be useful for removing haemolyzed and spilled protoporphyrin in the blood. However, it was not effective in patient No. 5 and 6, suggesting that sufficient protoporphyrin could not be removed. Therefore, plasmapheresis was followed by phlebotomy in these patients^15–17^. In patient No. 1, phlebotomy was introduced from the beginning. Phlebotomy is theoretically suitable for the removal of protoporphyrin in erythrocytes. However, hypermyelination due to the progression of anemia caused by phlebotomy may induce further production of protoporphyrins, which may worsen the condition. Therefore, we carefully performed a 400-mL phlebotomy for the first time by monitoring the blood data (such as Hb levels) and then slowly performed a 200-mL phlebotomy once every 1–2 weeks. After phlebotomy, liver damage rapidly subsided simultaneously with the decrease in protoporphyrin in all three patients; liver injury relapse was not noted thereafter. Phlebotomy has been proposed for congenital erythropoiesis (Günter's disease), acute liver and cutaneous porphyria^18^. This strategy is expected to suppress heme biosynthesis through the regulation of ALAS, a pathway restriction enzyme that leads to the accumulation of porphyrins. Although the effectiveness of phlebotomy in EPP patients has been shown this time, it is necessary to investigate the mechanism such as the regulatory action of ALAS in the future.

There are also reports on the onset and exacerbation factors of liver damage in EPP patients.

It has been reported that PP has low water solubility, which is an important mechanism for the onset of liver damage; the excretion of PP into the bile duct causes inflammation due to viscosity^19^. Furthermore, it has been reported that PP level and liver damage are correlated^4^. However, there are many cases in which the PP level does not correlate with the severity of liver damage, and therefore, it is necessary to analyze the mechanism of onset and severity of liver damage. In this study, we focused on ABCG2, a type of hepatocyte transporter, because it is involved in porphyrin transport^20–22^. We have previously reported the development of different levels of liver damage between siblings with the same PP level and the association between ABCG2 staining and liver damage^5^.

In this study, we increased the number of patients and conducted additional studies. Liver biopsies were performed in 3 of the 7 patients (No. 2, 5, and 6) to examine ABCG2 expression. ABCG2 staining was lower in patients No. 5 and 6 who had more severe liver damage than in patients No. 2. However, we also considered the possibility that the decreased expression of ABCG2 was affected by reactive oxygen species production due to the accumulation of protoporphyrin in hepatocytes. To solve this problem, we performed double staining with cadherin, which is a tight junction. Patient No. 5 maintains cadherin expression in protoporphyrin-deposited hepatocytes, indicating a selective loss of ABCG2 in the conserved cell membrane of hepatocytes. From this result, the accumulation of porphyrins in hepatocytes with loss of ABCG2 may be important for the onset and exacerbation of liver damage. However, we have not directly proved the causal relationship between the decreased expression of ABCG2 and liver damage, and further investigation is required in the future.

In recent years, there have been very interesting reports that ABCG2 deficiency protects against EPP-related hepatotoxicity^23^. It is considered that one of the mechanisms of liver damage protection is that the excretion of PP into the bile duct is reduced due to the decreased expression of ABCG2. On the other hand, in actual human patients, PP accumulation in hepatocytes and apoptosis of hepatocytes with decreased ABCG2 expression can be observed, so there may be a mechanism different from that of the mouse model. In any case, further research is needed on mechanism analysis.

In conclusion, phlebotomy has proven to be an effective treatment option in EPP patients with severe liver damage. Further research is needed to elucidate the pathophysiology of EPP in order to suppress porphyrin production and improve liver damage.

## Methods

### Patient background

We examined seven patients (No. 1–7) who were diagnosed with EPP and liver damage.

### Ethics declarations

Written informed consent were obtained from all of the patients enrolled in this study and ethical permission of this study was granted by the Review Boards of Kindai University Faculty of Medicine (approval number 25-085). All experiments were performed in accordance with the Declaration of Helsinki.

#### Pattern classification of liver damage

The pattern classification of liver damage was performed using the drug-induced liver damage diagnostic criteria (JDDW2004)^14^. Hepatocellular injury type; ALT > 2N + ALP ≤ N or ALT ratio/ALP ratio ≥ 5, cholestatic type; ALT ≤ N + ALP > 2 N or ALT ratio/ALP ratio ≤ 2, mixed type; ALT > 2 N + ALP > N and 2 < ALT ratio/ALP ratio < 5 (N: Upper limit of normal, ALT ratio = ALT value/N, ALP ratio = ALP value/N).

### Phlebotomy

Phlebotomy was first performed by removing 400 mL and then by removing 200 mL several times, depending on the patient's condition.

### Histological analysis

Percutaneous liver biopsy was performed in four patients. Liver biopsy samples were subjected to hematoxylin and eosin (H&E) staining. Immunohistochemical analyses were performed using mouse anti-human adenosine triphosphate-binding transporter G2 (ABCG2) antibody (Abcam, Cambridge, United Kingdom, BXP-21) and rabbit anti-human E-Cadherin (Cell Signaling Technology, Danvers, MA, 24E10). For fluorescence staining, secondary antibodies labeled with Alexa 488 or Alexa 555 were used (Life Technologies, Carlsbad, CA). Fluorescent images were taken using a confocal laser microscope (Carl Zeiss GmBH, Jena, Germany).

## Data Availability

All data generated or analyzed during this study are included in this article. Further inquiries can be directed to the corresponding author.
